# Vascular risk factors and Alzheimer’s disease

**DOI:** 10.1186/s12916-014-0218-y

**Published:** 2014-11-11

**Authors:** John T O’Brien, Hugh S Markus

**Affiliations:** Department of Psychiatry, University of Cambridge, Cambridge Biomedical Campus, Cambridge, UK; Department of Clinical Neurosciences, University of Cambridge, Cambridge Biomedical Campus, Cambridge, UK

**Keywords:** Alzheimer’s disease, Vascular risk, Stroke, Dementia, Risk factors, Prevention, Treatment, Hypertension

## Abstract

Vascular factors are now established risk factors for cognitive decline, both for dementia and its two main subtypes: Alzheimer’s disease (AD) and vascular dementia. Their impact likely goes beyond causing an increase in concurrent vascular pathology, since they have been associated with increasing the risk of degenerative Alzheimer (plaque and tangle) pathology, either by increasing its rate of formation or reducing elimination from the brain, or a mixture of the two. A comprehensive series of reviews published in *BMC Medicine*, investigates the relationship between AD and cardiovascular diseases and risk factors from a clinical, pathological and therapeutic perspective. Whilst links between vascular factors and AD have clearly been demonstrated at both the clinical and pathological level, the nature of the relationship remains to be fully established and there is a lack of high quality treatment studies examining the extent to which vascular risk modification alters AD disease course. Further longitudinal mechanistic and therapeutic studies are required, especially to determine whether treatment of vascular risk can prevent or delay the onset of AD and/or reduce its rate of clinical progression.

## Editorial

Dementia is a large and growing public health burden, affecting an estimated 35 million people worldwide with a prevalence that will double in the next 25 years. Not surprisingly, it is at the top of governmental priorities for service development and research, as evidenced by the recent international governmental G8 summit on dementia in 2013 and the publication of dementia plans within many countries. Alzheimer’s disease (AD) remains the most common cause, responsible for around two-thirds of all cases, the other major causes being vascular, Lewy body and fronto-temporal dementias. Treatments for AD, which provide symptomatic transmitter replacement in the form of cholinesterase inhibitors and memantine, have been available for the last 15 years, but these provide at best modest clinical efficacy, and none convincingly modify underlying disease course. Since their development almost 20 years ago, there have been major advances in our understanding of the molecular pathology of AD [1]. Most research has focused on the overproduction and abnormal deposition of amyloid (especially ABeta42) and tau (especially phospho-tau), the two proteins which comprise the recognised pathological hallmarks of the disease, senile plaques and neurofibrillary tangles. Several therapeutic attempts to affect underlying disease processes, based on modifying deposition and/or clearance of amyloid or tau, are underway, although unfortunately initial clinical trials, largely around reducing deposition of amyloid, have been largely negative [2]. This, together with findings from recent genome wide association studies which have identified a number of risk genetic variants with potential influences on vascular and inflammatory mechanisms [3], has emphasised not only that the full etiological pathway for AD remains to be elucidated, but that other mechanisms may be equally or more important than abnormal protein deposition in terms of providing potentially tractable therapeutic targets. Vascular factors and mechanisms have emerged as an area of key importance.

It has long been recognised that there is a close relationship between AD and vascular factors [4]. Originally, this was recognised as a pathological overlap, with an increased prevalence of vascular lesions – especially white matter lesions and lacunes – and the finding that these lower the threshold for the clinical expression of dementia at a certain burden of Alzheimer pathology [5,6]. However, it now appears that the relationship goes much deeper than this. Vascular risk factors, including hypertension, smoking and hypocholesterolemia are now recognised clinical risk factors not just for the clinical diagnosis of AD, but for the presence of Alzheimer pathology [7]. It has also been demonstrated that Alzheimer pathology in the form of cerebral amyloidosis can affect vascular and endothelial function, which may further impair vascular mechanisms and, potentially, the elimination of abnormal proteins, such as amyloid, from the brain [8]. In this timely series of comprehensive review articles the complex and important relationship between vascular disease and AD are explored in more detail, from a pathological, clinical and therapeutic perspective.

Jellinger and Attems [9] provide a detailed account of the overlap between AD and vascular disease from a pathological perspective. They highlight that while a close pathological overlap is well recognised, several key questions remain unresolved. It is recognised that the vast majority of (but not all) Alzheimer cases also have cerebral amyloid angiopathy, and that co-existent cerebrovasular lesions, primarily subcortical white matter lesions or lacunes, are common. However, there is not a good correlation between the two (vascular and degenerative pathology) in anatomical co-location, nor is it clear whether increasing vascular pathology is associated with increasing severity of degenerative pathology. Evidence to date is reasonably consistent in showing that minor degrees of vascular pathology, usually subcortical, are important in lowering the threshold at which a certain degree of Alzheimer pathology is associated with dementia. However, in more severe AD cases it appears that the Alzheimer pathology predominates in terms of an impact on cognition, and cases of ‘mixed dementia’ more often involve large vessel vascular pathology, such as cortical infarcts, rather than small vessel disease. This has very substantial clinical relevance, in that whilst vascular changes may play a key role in bringing forward the presentation of AD in those with mixed pathology, the presence of minor degrees of vascular pathology in established AD is unlikely to be of clinical significance.

De Bruijn and Ikram [10] discuss the relationship between both cardiovascular disease and risk factors and AD from a clinical perspective. They find good evidence for the relationship between stroke, atrial fibrillation and coronary heart disease and AD. Stroke is a well recognised risk factor for dementia, including AD, although the authors highlight that the relationship between the two remains unclear and may involve both lowering the threshold for the expression of dementia in those with degenerative pathology (as discussed by Jellinger and Attems [9]) as well as cerebrovascular disease directly accelerating amyloid production or decreasing its clearance. Several markers of vascular disease are also associated with increased AD risk, including intima media thickness, carotid plaque severity, and large vessel calcification as measured by computed tomography (CT) scanning. There is a stronger relationship between markers of small vessel disease, including lacunes and white matter lesions, and AD risk - very much mirroring the pathological overlap described by Jellinger and Attems. In addition, imaging has highlighted the potentially deleterious impact of cerebral microbleeds for both cognitive impairment and incident AD. This link has biological plausibility, since both are associated with cerebral amyloidosis, but as cerebral microbleeds often occur at an increased rate concurrently with white matter lesion burden and lacunes, teasing out the specific individual contributions of different types of subcortical vascular pathology remains very challenging, especially as they are measured in different ways [11]. In terms of cardiovascular risk factors, De Bruijn and Ikram [10] summarise evidence for the increasingly robust relationship between several risk factors including hypertension, diabetes, hypercholesterolemia, smoking and obesity and AD, whilst also suggesting a possible beneficial or protective effect of a ‘Mediterranean’ diet and physical exercise. However, relationships are complex. For example, while mid-life hypertension has been shown to be a risk factor for AD, the opposite is true in later life where hypotension in later life is related to AD, an inconsistency most likely explained by the effects of Alzheimer’s pathology or dementia in reducing blood pressure close to the time of disease onset. There remains controversy as to whether raised homocysteine levels are a risk factor for AD. Raised homocysteine has been consistently associated with increased vascular risk, and has been associated with brain atrophy and tangle pathology in some studies, but there are discrepant findings. This relationship is particularly important to determine since raised homocysteine levels were associated with increased brain volume loss on serial magnetic resonance imaging (MRI) in those with mild cognitive impairment in one study [12], and reducing homocysteine levels through folate and vitamin B12 replacement has been advocated as a potential treatment for AD. The authors also highlight emerging risk factors, in particular inflammation, chronic kidney disease and hypothyroidism, all conditions which may be associated with altered amyloid processing.

Finally, Valenti *et al.* [13] address perhaps the most critical and clinically important question, which is, given the clear overlap between vascular factors and AD, does the treatment of vascular risk in those with AD provide any benefit, either in terms of improvement or, more likely, decreasing the rate of subsequent cognitive decline? Perhaps surprisingly, the answer to this question is far from clear, despite the fact that the authors review 14 observational studies and 11 randomised controlled trials. Some observational studies suggest that enhanced treatment of vascular risk using a multifactorial approach, including the treatment of hypertension and statin therapy, may improve cognitive outcomes, but they all suffer from potential bias. Randomised controlled trials are relatively few in number, often of modest size with a short duration of follow-up which, while appropriate for cardiovascular outcomes, is insufficient for determining longer term cognitive outcomes. Indeed, several of the studies were primarily set up to assess cardiovascular outcomes, and when these end points were met the trials were stopped [14]. The only randomised controlled trial examining the effectiveness of a vascular care package compared to placebo did find significant differences between groups in the expected direction in terms of lower homocysteine and cholesterol in the actively treated group, but no impact on cognitive end points [15]. There have been surprisingly few controlled studies in other areas – none of antihypertensive treatment compared with placebo in established AD and several small and one large trial of a statin [16], which did not find a benefit of atorvastatin compared to placebo in a 72 week study of those with mild to moderate AD. The authors conclude that until the evidence base is stronger it remains a reasonable option to treat vascular risk factors in AD, particularly in those with co-existent cardiovascular disease, a view supported by consensus Guidelines [17], but the authors call for large adequately powered trials, both in mild cognitive impairment and the early stages of AD, to determine whether treating vascular risk factors delays progression in those without overt cerebrovascular disease. As guest editors of this series of articles, we very much concur with this view and also with Valenti *et al’s* [13] conclusion that the outcomes of such studies would potentially have a major impact on the way AD is assessed and treated, both in primary and secondary care.
